# Development and evaluation of educational activities for medical students and pediatricians about climate and health

**DOI:** 10.3389/fpubh.2026.1802368

**Published:** 2026-04-14

**Authors:** Nicholas C. Newman, Jacqueline M. Knapke, Shiu-lin Tsai, Reid Hartmann

**Affiliations:** 1 Department of Pediatrics, University of Cincinnati College of Medicine, Cincinnati, OH, United States; 2 Environmental Health and Lead Clinic, Division of General and Community Pediatrics, Cincinnati Children's Hospital, Cincinnati, OH, United States; 3 Department of Environmental and Public Health Sciences, University of Cincinnati College of Medicine, Cincinnati, OH, United States; 4 Department of Family and Community Medicine, University of Cincinnati College of Medicine, Cincinnati, OH, United States; 5 Department of Pediatrics in Emergency Medicine, Columbia University at Irving Medicine Center, New York, NY, United States

**Keywords:** climate, climate health, environmental health, medical education, medical student, pediatrics

## Abstract

**Introduction:**

The medical profession is starting to incorporate climate change and health into the education for physicians. Increased awareness of the health impacts of a warming planet has made this an important topic to teach pediatricians and medical students; however, most have no previous background in environmental health.

**Curriculum design:**

We developed didactic lectures on climate change and health customized for medical students and pediatricians that included clinical vignettes on climate-related health topics. Topics included the health effects of flooding, heat, poor air quality, and changes in insect vector distribution. Lectures were presented to 1st year medical students during a class regarding social influences on health and to pediatricians at a national meeting. An anonymous, post-presentation questionnaire was administered online, accessible to attendees through a QR code displayed at the end of the lecture. It assessed knowledge and perspectives regarding climate change and health, and the attendees' rating of the effectiveness of the presentation.

**Results:**

There were 81 (47% of 172 attendees) first year medical students and 13 (26% of 50 attendees) pediatricians who completed the questionnaire. From the questions regarding the climate and health knowledge assessment, the medical students scored 95.1% correct overall and the pediatricians scored 63.5% correct overall (*p* < 0.01). For assessing perspectives regarding climate change, we used the previously validated Global Warming's Six Americas audience segmentation (Six Americas Super Short Survey). 93.6% of medical students and 92.3% of physicians were either “Alarmed” or “Concerned” about climate change. Both students and pediatricians reported satisfaction with the session overall, 8.9/10 and 8.2/10, respectively.

**Discussion:**

Both medical students and pediatricians reported high levels of concern about climate change. Knowledge regarding climate change and health was lower overall for pediatricians compared with medical students. To address knowledge gaps, future climate change and health education should be tailored to the learning needs and styles of practicing pediatricians.

## Highlights

- Changes in the climate are impacting and will impact human health, but integrating these concepts into medical education are happening slowly
- We developed and evaluated a climate and health presentation that was used for first year medical students and practicing pediatricians
- Both groups were concerned about the impacts of climate change, but have different learning needs

## 1 Introduction

The medical profession is starting to grapple with climate change as a threat to human health. The 2023 Lancet Countdown called for an increase in capacity for health systems to respond to climate-related health problems and for a transition to zero emission health systems (1). Changes in precipitation, temperature, composition of air pollution, water quality, disease vector geography, and whole ecosystems are affecting human health and are predicted to pose huge risks to human health in the future, particularly children (2). The health sector contributes approximately 8.5% of greenhouse gas emissions in the U.S., but only 12.4% of large children's hospitals are publicly tracking climate change mitigation efforts (3). On the other hand, both pediatric program directors and trainees agree that climate change and health is an important topic, but only 17% of program directors surveyed felt very or moderately knowledgeable about climate change and health impacts (4). This emphasizes the importance of building additional practitioner competency in this area (5). Medical students have been active in calling for climate change and sustainability to be added to the medical school curriculum, resulting in medical school electives being created at some medical schools (6–9). However, creating an elective may unintentionally exclude students who cannot attend and may also self-select the students with an interest in the topic. Incorporating content into existing and required classes would increase medical students' exposure to the health impacts of climate change. In addition, most physicians in practice also need this training; incorporating content on climate change and health through existing, well-attended, national conferences would be one approach.

Basing our approach on adult learning theory, we addressed this educational gap by (1) developing didactic lectures on climate change and health customized for medical students and pediatricians, and (2) studying the impact of the lectures on attendees. This paper describes an approach to incorporate climate change and health didactic content into medical education for first-year medical students and practicing pediatricians. We evaluated this approach to understand attitudes and learning needs for each group and compared the results between the groups to inform the development of future educational materials. The project was developed as part of the work of the Pediatric Environmental Health Specialty Unit (PEHSU) network to provide expertise in education and outreach in environmental health (10).

## 2 Curriculum design

In response to requests from previous classes of medical students to better include climate change and health into the medical school curriculum, we developed a 2-h session for 1st year medical students that was incorporated into a required class, “Physician and Society” at the University of Cincinnati. This course introduces medical students to social determinants of health and helps develop their physician identity in the context of the community. The session consisted of a 1-h lecture followed by a panel discussion with an environmental epidemiologist, clinician, and hospital sustainability professional. The topics for the lecture included a discussion of the basic mechanisms of climate change, healthcare-related greenhouse gas emissions, and clinical vignettes describing climate-sensitive health conditions such as flooding, air pollution, urban heat, and air pollution. Similarly, there was a request for a 1-h lecture regarding climate change and children's health that came from the American Academy of Pediatrics Section on Integrative Medicine for their national meeting, so the materials created for the medical students was adapted for use with pediatricians in practice. These topics and approach were chosen to align our lecture with adult learning theory, as best could be done based on the structure of the class and national meeting's didactic structure. All lectures were done in person.

Using Kern's Six Steps to Curriculum Development as a guide, the presentation and the evaluation were designed for medical student and pediatrician audiences (Table 1) (11). The survey was designed to assess Level 1 (reaction) and Level 2 (learning) from the Kirkpatrick Model of training evaluation. The medical student survey contained 16 questions, and the pediatrician survey contained 14 questions. The questions from both surveys are included in the Supplementary material. To evaluate the presentation, participants accessed an online survey through a QR code on the last slide of the presentation; participation was voluntary and anonymous. These questions were developed for these activities and had not been piloted or validated. Since this was our first attempts at this activity, we did not have an *a priori* estimate of the difficulty of the questions.

**Table 1:** Design of climate change and health educational presentation.

Kern's design step	Specific data and actions for this project
Problem identification and general needs assessment	Climate change is an important threat to public health - Physicians lack formal training in most environmental health issues, including climate change and health
Needs assessment of targeted learners	No current course content at our medical school that addresses climate change and health - Medical students requested course content in climate change and health - Leadership at AAP Sections requested content on climate change and health
Goals and objectives	Through participation in the presentation, attendees will be able to: - Define climate change and describe basic mechanisms responsible for a changing climate - Describe health impacts from climate change - Review actions that promote climate change adaption and mitigation in clinical settings to help our patients
Educational strategies	PowerPoint presentation presented as part of existing educational venues (lecture in medical school course, lecture at pediatric national meeting)
Implementation	Presentation consisted of: - General introduction to climate change and its mechanisms - Discussion of healthcare sector's contribution to greenhouse gas emissions - Clinical vignettes illustrating several health effects from climate change (extreme heat, acclimatization, extreme weather, water-borne illness, poor air quality, vector ecology, vector-borne disease)
Evaluation and Feedback	Post-presentation survey to evaluate knowledge, instructional methods, and attitudes toward climate change Survey was designed to provide immediate feedback and knowledge-based assessment

The medical student presentation took place in August 2023 during the first hour of a 2-h session of a required course, “Physician and Society” at the University of Cincinnati. The pediatricians were attendees at an hour-long presentation on climate change and health which took place at the joint session of the Section on Osteopathic Pediatricians and the Section on Integrative Medicine at the American Academy of Pediatrics 2023 National Conference and Exhibition.

### 2.1 Statistical analysis

Descriptive statistics (mean, standard deviation, counts) for quantitative survey responses were calculated. Responses were compared between groups (medical students, pediatricians) using either *t*-test (mean scores on knowledge assessment questions, satisfaction score), Fisher's exact test (categorical responses), or contingency tables as appropriate. All calculations were done using Microsoft Excel (Excel for Office 365, Microsoft, Redmond, WA) or R 4.4.1 (R Foundation for Statistical Computing, Vienna, Austria) package Rcmdr 2.9-2 (12). Cohen's d_s_ was calculated to estimate the effect size for difference in knowledge assessment score between medical students and pediatricians based on the method described by Lakens (13).

Qualitative survey responses were analyzed using a thematic analysis approach where data were coded in order to construct thematic patterns across participant responses, with the intent of comparing and contrasting themes between the two groups (14).

Audience segmentation for the Six America's Short Survey (SASSY) questionnaire, a tool to gauge perspectives about climate change, was calculated using the SASSY web-based scoring tool (https://climatecommunication.yale.edu/visualizations-data/sassy/) (15).

The Cincinnati Children's Hospital Institutional Review Board reviewed the project and determined that it was not human subjects' research.

## 3 Results

### 3.1 Quantitative results

**Figure 1:**
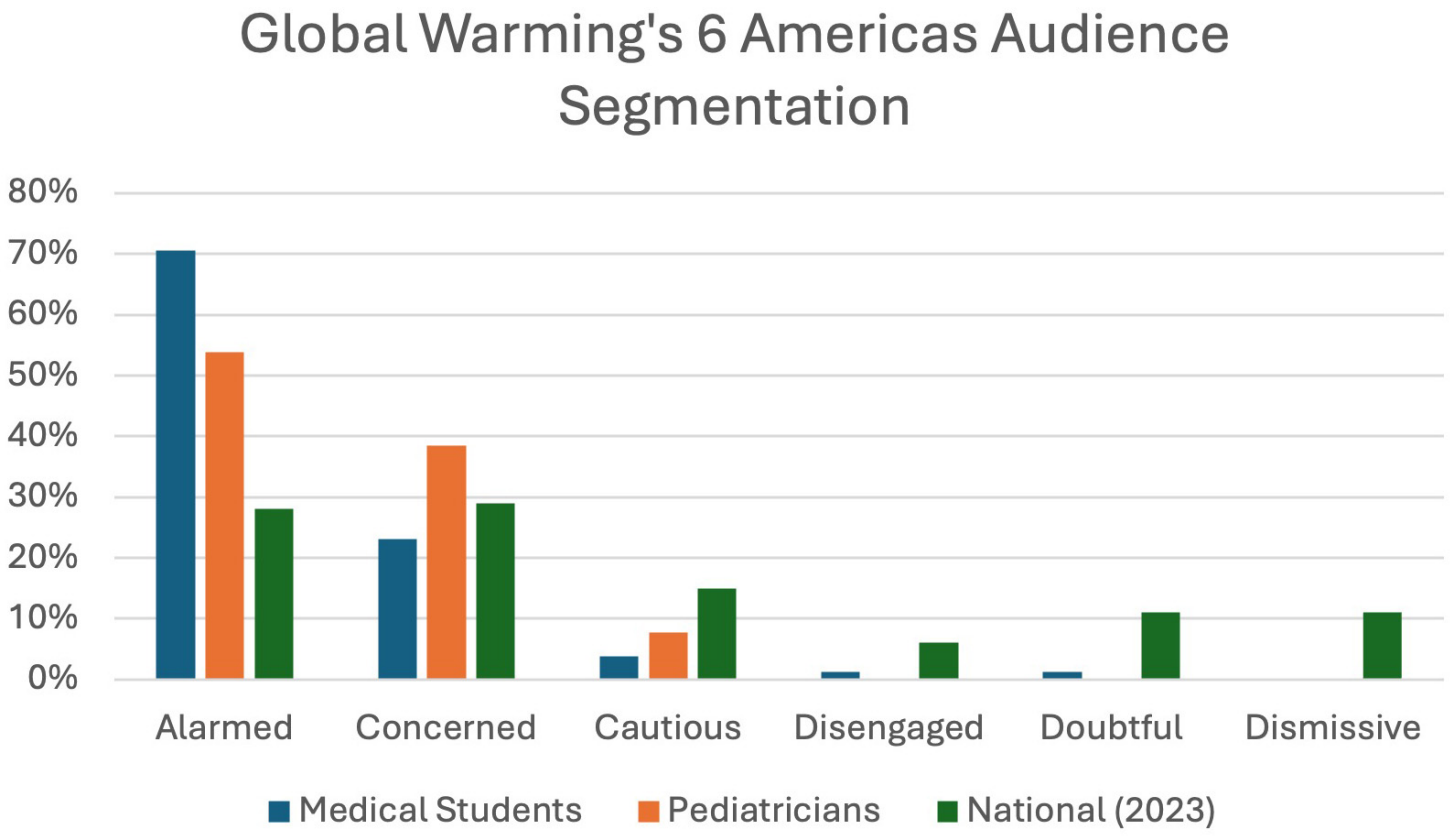
Perspectives regarding climate change for 1st year medical students and pediatricians with national results as reference (Six Americas Super Short Survey results).

There were 81 medical students, 47% of 172 in the class, and 13 pediatricians, 26% of the 50 attendees at the joint session, who completed the post-presentation survey. For the knowledge assessment section, the medical students scored 95.1% correct overall and the pediatricians scored 63.5% correct overall, *p* < 0.0001, Cohen's d_s_ 2.4. Examining the scores for each of the identical questions (1–3), the pediatrician group did more poorly on the questions related to the basics of climate change and healthcare sector contribution of greenhouse gasses as compared with the medical students (Table 2). Both the medical students (97.5%) and pediatricians (100%) reported that the session achieved the learning objectives. Medical students reported that the session was the perfect length (93.6%) vs. only 61.5% of the pediatricians. Pediatricians reported that the session was too long (15.4%) or too short (23.1%). SASSY audience segmentation indicated that 70.5% of the medical students were “Alarmed” about climate change, whereas 53.8% of the pediatricians were “Alarmed” about climate change (Figure 1). Descriptive and inferential statistics can be found in Table 2.

**Table 2:** Results from post-presentation survey, broken down by learner group (medical students or pediatricians).

Variable	Medical students	Pediatricians	*p*-value	Note
Knowledge assessment				
Mean (SD) score on knowledge assessment	95.1 (10.0), *n* = 81	63.5 (25.6), *n* = 13	< 0.001	*t*-test
Question 1 correct	97.5%	69.2%	< 0.01	Fisher's exact test
Question 2 correct	100%	92.3%	0.01	Fisher's exact test
Question 3 correct	98.8%	46.2%	< 0.001	Fisher's exact test
Question 4 correct	84.0%	46.2%		Not compared, questions different
Lecture feedback				
Lecture achieved learning objectives well or very well^*^	97.5%	100%	0.2	Chi-squared test, *n* = 78 students, *n* = 13 pediatricians
Session was perfect length^*^	93.6%	61.5%	< 0.01	Fisher's exact test
Satisfied with session, mean (SD) of 1–10 scale^*^	8.9 (1.2)	8.2 (1.3)	0.08	*t*-test
Climate change attitudes—Six America's Short Survey (SASSY) audience segmentation				
SASSY segment^*^			0.49	Overall scores, Fisher's exact test
Alarmed	70.5%	53.8%		
Concerned	23.1%	38.5%		
Cautious	3.8%	7.7%		
Disengaged	1.3%	0%		
Doubtful	1.3%	0%		
Dismissive	0%	0%		

^*^78 students responded to these questions.

### 3.2 Qualitative results

Fifty-four participants (46 medical students and 8 pediatricians) responded to three open-ended questions in the post-training survey. Several themes were shared between both groups. Participants reported that instructional methods that were very applied were most effective (e.g., case studies that demonstrated the real-world health impacts of climate change, evidence-based methods for understanding climate change such as the air quality index). Many participants desired a greater focus on the impacts of climate change (e.g., global migration) and wished that presentation materials such as slides, and other resources could be made available before or after the talk. Both groups expressed negative emotional reactions to the content such as sadness and dread. Related to this negative emotional reaction, participants expressed a desire for more actionable information: medical students wanted information on volunteer or advocacy opportunities related to climate change, and pediatricians wanted information on how best to educate colleagues and how best to address patient issues related to climate change. Additional themes were present only in the medical student group, likely because of the larger sample size. Medical students appreciated that the slides and presentation style were concise and engaging, and they would like more educational sessions on this topic.

## 4 Discussion

In our sample of medical students and pediatricians attending a lecture on climate change and health, we demonstrated significant differences in knowledge of the subject matter by group. Medical students scored higher on the knowledge assessment compared to clinicians in practice (95.1 vs. 63.5%). During the lecture neither group was informed of the upcoming feedback questionnaire. A higher proportion of medical students (70.5%) classified as “Alarmed” in the audience segment analysis based on the global warming's “Six Americas Short Survey” compared with pediatricians (53.8%). Both of our groups are more alarmed about climate change than a nationally representative survey carried out during the same time period where 28% of adults were “Alarmed” (16). Based on the free text comments on the assessment, both groups appreciated the case vignettes in the presentation and explanation of the Air Quality Index and both groups wanted more to hear about more actionable steps that they could take.

Our previous work (17) suggests that pediatricians in practice may learn better through non lecture approaches. It is important to reach this group as they comprise a larger segment of the pediatric workforce compared to newly trained pediatricians or medical students. One of the challenges is that for physicians currently in practice, their knowledge of environmental health concepts or climate and health may be limited as more immediately relevant clinical concerns are front of mind (5, 18). A study in a large hospital in Germany indicated that healthcare workers considered climate change mitigation as important but were concerned about how this could interfere with patients' health (18). Although lecture format is the mainstay of large medical meetings, it may not be the best way to engage and educate adult medical learners; modifications could increase engagement (19).

### 4.1 Limitations

There are several limitations to our project. Since the assessment tool was meant to assess cross-sectional knowledge and opinions, there was no pre-test of climate change and health knowledge done, so we cannot rigorously assess knowledge gained from participating in the activity. Additionally, the difference in the scores of the knowledge assessment between groups may be a function of an age cohort effect as medical students are very engaged about climate change as compared with other medical groups (6). Although the climate and health content of the two presentations was nearly identical, the presentation for pediatricians had additional slides regarding general concepts in pediatric environmental health. The response rate between both groups was low (45% for medical students and 26% for pediatricians) therefore may not be representative of all the attendees present at the lectures and could introduce bias. Additionally, neither group may be representative of the larger group of medical learners. Specifically, the pediatricians self-selected to attend the climate change and health lecture, and this could introduce bias in their attitudes regarding climate change. A strength is that the lectures were designed to illustrate the effects of climate change on health using clinical vignettes and were designed and presented by a physician who is formally trained in pediatrics, environmental medicine, and climate change and health.

### 4.2 Future directions

In the future, we plan to incorporate an assessment of prior knowledge of climate change and health with a less dense lecture format incorporating interactive “breaks” to aid with learner engagement. Fine tuning the education to the specific climate change related health impacts expected in a clinician's patient population (extreme weather, flooding, wildfires, heat stress, etc.) using clinical examples may also increase engagement. Finally, to address the emotional reaction to the material (sadness and dread) expressed by some learners, future education should include specific action steps to take to address climate and health impacts of healthcare operations that would improve patient care, such as reducing unnecessary pharmaceutical use. Climate change is a huge problem that impacts health in multiple ways. Continuing medical education will need to integrate climate and health considerations across relevant clinical scenarios and present evidence-based treatments and health promotion recommendations.

## 5 Conclusions

In this small project, both medical students and pediatricians in practice report concern regarding climate change. A climate change and health teaching activity for medical students and pediatricians was well-received by both learner groups. Differences in knowledge regarding climate change and health identified between the groups suggests that additional education and new approaches to integrate climate and health concepts into clinical actions will be needed for practicing pediatricians.

## Data Availability

The raw data supporting the conclusions of this article will be made available by the authors, without undue reservation.
