# Supplementary material for: Development and evaluation of educational activities for medical students and pediatricians about climate and health
Source: Front Public Health. 2026 Apr 14;14:1802368. doi: 10.3389/fpubh.2026.1802368 (PMC13121261; doi:10.3389/fpubh.2026.1802368)
Supplement: Supplementary file 1 [file Data_Sheet_1.pdf]

Supplemental Material:

Questions from post-presentation survey. Possible responses are in square brackets. Except for question 4, the possible responses were the same.

Question Number	Medical Students	Pediatricians
1	Climate is the long-term average weather at a specific location. [True/False]	Climate is the long-term average weather at a specific location.
2	Which of the following groups is most vulnerable to the effects of climate change? (please select the most appropriate response) [Older adults, Low income communities, Children, Communities of color, All of the above, None of the above]	Which of the following groups is most vulnerable to the effects of climate change? (please select the most appropriate response)
3	The percentage of US greenhouse gas emissions from the health care sector is approximately [1.5%, 4.4%, 8.5%, 12.1%]	The percentage of US greenhouse gas emissions from the health care sector is approximately
4*	Acclimatization (physiological adaptation) to heat results in higher sweat rate and expansion of blood volume. These changes occur over the span of [Minutes, Hours, Days, Weeks]	Based on the presentation, increased incidence of gastroenteritis in children was observed for children living within what distance of the combined sewer overflow? [5 kilometers, 500 meters, 100 meters, 50 meters]
5^	How important is the issue of global warming to you personally? [Extremely important, Very important, Somewhat important, Not too important, Not at all important]	How important is the issue of global warming to you personally?
6^	How worried are you about global warming? [Very worried, Somewhat worried, Not very worried, Not at all worried]	How worried are you about global warming?
7^	How much do you think global warming will harm you personally? [A great deal, A moderate amount, Only a little, Not at all, Don't know]	How much do you think global warming will harm you personally?
8^	How much do you think global warming will harm future generations of people? [A great deal, A moderate amount, Only a little, Not at all, Don't know]	How much do you think global warming will harm future generations of people?
9	How well do you think that the Climate Change Session addressed the Learning Objectives? [Very well, Well, Neutral, Poorly, Very Poorly]	How well do you think that the Climate Change Session addressed the Learning Objectives?
10&	Were the reading materials for this session helpful? [Yes, No, Don't know/didn't read materials]	

11	How did you find the length of the session? [It was too long, It was too short, If was the perfect length]	How did you find the length of the session?
12&	Do you think the session was well organized? [Yes, No]	
13	What did you like least about the session? [Free text response]	What did you like least about the session?
14	What did you like most about the session? [Free text response]	What did you like most about the session?
15	How satisfied are you with the session? [0-10 scale, 0=very unsatisfied, 10=very satisfied]	How satisfied are you with the session?
16*	Do you have any suggestions for us to improve future pediatric environmental health and climate change and health sessions? [Free text response]	Do you have any suggestions for us to improve future climate change and health sessions? [Free text response]

* Different question for each group
^ SASSY (Six Americas Super Short Survey) question
& question not included on pediatrician's survey
